# Only 26% of Native Knees Show an Identical Coronal Functional Knee Phenotype in the Contralateral Knee

**DOI:** 10.3390/jpm14020193

**Published:** 2024-02-09

**Authors:** Manuel-Paul Sava, Alexandra Leica, Felix Amsler, Sotirios Leles, Michael T. Hirschmann

**Affiliations:** 1 Department of Orthopaedic Surgery and Traumatology, Kantonsspital Baselland (Bruderholz, Liestal, Laufen), CH-4101 Bruderholz, Switzerland; 2 Department of Clinical Research, Research Group Michael T. Hirschmann, Regenerative Medicine & Biomechanics, University of Basel, CH-4001 Basel, Switzerland; 3 Amsler Consulting, Gundeldingerrain 111, CH-4059 Basel, Switzerland; 4 Iatriko Athinon Clinic, Distomou 5–7, 15125 Marousi Attica, Greece

**Keywords:** knee, native alignment, coronal alignment, sagittal alignment, axial alignment, left-to-right symmetry, correlations, systematic differences, 3D, SPECT/CT, functional knee phenotype, laterality

## Abstract

Background: A comprehensive exploration evaluating left-to-right knee symmetry across all anatomical planes utilizing three-dimensional (3D) scans stands absent from the existing body of research. Therefore, the primary objectives of this investigation involved examining potential differences and resemblances in alignment and structure between left and right non-osteoarthritic (native) knees in various planes (coronal, sagittal, and axial) using three-dimensional single-photon emission computed tomography/computed tomography (SPECT/CT) images. Methods: A total of 282 native knees from 141 patients were retrospectively gathered from the hospital’s records. Patients, aged between 16 and 45, who underwent Tc99m-methyl diphosphonate SPECT/CT scans for both knees, adhering to the Imperial Knee Protocol, were included. A statistical analysis was conducted, including 23 knee morphometric parameters, comparing left and right knees, and classifying them based on functional knee phenotypes across the coronal, sagittal, and axial planes. Results: Regarding the functional coronal knee phenotype, 26% of patients (n = 37) exhibited identical phenotypes in both knees (*p* < 0.001). Significant correlated similarities between the left and right knees were observed in the coronal plane (Pearson’s r = 0.76, 0.68, 0.76, 0.76, *p* < 0.001) and in several morphometric measures in the sagittal plane (Pearson’s r = 0.92, 0.72, 0.64, *p* < 0.001). Moderately correlated similarities were noted in the axial plane (Pearson’s r = 0.43, 0.44, 0.43, *p* < 0.001). Conclusions: Only 26% of native knees exhibit an identical coronal phenotype in their contralateral knee, whereas 67% have the adjacent coronal phenotype. Strongly correlated resemblances were established across various left and right knee morphometric parameters in the coronal, sagittal, and axial planes. These findings could enhance decisions in procedures like total knee arthroplasties or osteotomies, where alignment is key to outcomes, and reveal a potential for future artificial intelligence-driven models to improve our understanding and improve personalized treatment strategies for knee osteoarthritis.

## 1. Introduction

The persistence of dissatisfaction rates among patients undergoing total knee arthroplasty (TKA) despite numerous advancements in orthopedics has prompted a reevaluation of the conventional mechanical alignment paradigm [1,2,3,4]. Recent inquiries into refining outcomes have challenged the long-standing gold standard of mechanical alignment in knee surgeries [5,6,7,8,9].

Hirschmann et al. catalyzed this discussion by introducing and applying a coronal functional knee phenotype classification, allowing for a meticulous evaluation of patient-specific knee anatomy [5,6,7,8]. The research revealed that knee phenotypes representative of mechanical alignment were notably scarce, identified in only 5.6% of men and 3.6% of women. Similarly, knee phenotypes indicative of anatomical alignment were found in just 18% of men and 17% of women [8]. However, a direct determination of native alignment and morphology from knees affected by osteoarthritis (OA) poses considerable challenges [5,9,10,11]. Exploring the existence of significant symmetry between knees on opposite sides has emerged as a potential simplification in pre-operative planning for TKA and realignment procedures [12]. Several studies, ranging from cadaveric investigations to imaging analyses, have suggested the likelihood of similarities in contralateral morphometric parameters within lower limbs [13,14,15]. Furthermore, insights from Nedopil et al. demonstrated that utilizing the alignment of the opposite knee as a reference for coronal alignment led to a substantial improvement in patient-reported function among kinematically aligned (KA) TKA patients [16]. Similarly, Mullaji et al. highlighted the reliability and validity of assessing lower limb alignment by comparing it with the unaffected limb on the opposite side [17]. Beckers et al., in contrast, observed no discernible symmetry between both knees in the coronal plane [18]. Amid these affirmations, the picture remains inconclusive. This disparity in findings underscores the complexity of ascertaining consistent bilateral knee alignment when discussing the coronal plane, where a consensus eludes researchers. Firstly, differences in imaging modalities used to assess coronal plane alignment can yield divergent results [19]. Additionally, variations in the assessment of knee morphometric parameters between standing and supine positions may also contribute to discrepancies [20]. Moreover, while some studies focus on healthy knees, the inclusion of individuals with underlying conditions or varying levels of joint health can further complicate interpretations [13,15,17]. Furthermore, factors such as age, gender, and biomechanical variations can also contribute to the observed variability in knee alignment studies [21]. Moreover, a noteworthy observation across these studies is the predominant focus on the coronal plane, with limited inclusion of analyses on the axial and sagittal planes, primarily owing to the prevalent use of long-leg radiographs [16,17,18]. Assessing axial and sagittal planes alongside the coronal one is crucial for producing a comprehensive evaluation and optimal outcomes in knee joint altering surgical interventions such as TKA and osteotomies. Understanding knee morphology in three dimensions allows for a more accurate alignment and biomechanical restoration, which are essential for long-term success and patient satisfaction [19,22]. Axial parameters, pertaining to torsion and rotation, influence patellofemoral tracking and stability, affecting postoperative function and implant longevity [22,23]. Sagittal parameters play a significant role in knee flexion, stability, and range of motion [24]. Neglecting these dimensions can lead to complications like instability, malalignment, and premature implant wear [25]. However, a comprehensive exploration evaluating left-to-right knee symmetry across all anatomical planes utilizing three-dimensional (3D) scans stands absent from the existing body of research. This significant research gap emphasizes the need for a comprehensive study probing into knee symmetry across all anatomical planes, employing advanced 3D scans. Such an investigation could potentially identify nuanced symmetrical patterns or disparities, offering insights into native knee morphology and alignment beyond the confines of the coronal plane. This expanded understanding could transform pre-operative planning strategies and enhance the precision of knee surgeries, ultimately improving patient outcomes and satisfaction in the realm of TKA and realignment procedures. 

Therefore, the aim of this present study was to check for potential differences and similarities in alignment and structure between the left and right non-osteoarthritic (native) knees in various planes (coronal, sagittal, and axial) using three-dimensional single-photon emission computed tomography/computed tomography (SPECT/CT) images. The hypothesis was that a strict symmetry between left and right native knees does not exist in any of the three anatomical planes. However, several morphometric parameters would display a high degree of left-to-right resemblance. 

## 2. Materials and Methods

This retrospective review encompassed a total of 282 knees without osteoarthritis (non-OA) from 141 patients, comprising a male-to-female ratio of 90:51. The average age, represented as mean ± standard deviation (SD) and range, stood at 30.1 ± 6.7 years, spanning between 16 and 44 years. Data for this analysis were sourced from a prospectively maintained hospital registry. Eligible subjects, aged between 16 and 45, underwent Technetium 99m-methyl diphosphonate (99mTc-HDP) SPECT/CT scans for both knees, adhering strictly to the Imperial Knee Protocol [26]. A comprehensive description of the SPECT/CT protocol and the software employed for planning (KneePLAN 3D, Symbios, Yverdon les Bains, Switzerland) has been previously documented [6]. Exclusion criteria involved patients with a history of knee, hip, or ankle prosthesis, prior osteotomies or fracture treatments, collateral ligament injuries, or radiological signs of osteoarthritis. Furthermore, individuals with knee flexion exceeding 15° in the SPECT/CT scan were also excluded from the study, as excessive knee flexion during scanning can introduce variability in knee alignment measurements, potentially skewing the results and compromising the accuracy of the analysis [25]. High levels of knee flexion can also lead to joint incongruity, making it challenging to accurately assess morphometric parameters and symmetry between knees [27]. 

The primary reasons for conducting SPECT/CT scans were diverse, with sports injuries accounting for the highest proportion (30.85%), followed by patella-related pathologies (12.41%), idiopathic knee pain (7.44%), and osteochondrosis dissecans (4.60%). The precision of the measurements undertaken, inclusive of inter- and intra-observer reliability, was previously established as excellent, displaying a measurement variability within 1° [6]. Statistical analyses encompassed a left-to-right comparison of 23 knee morphometric parameters from 141 patients, alongside their cataloguing in accordance with Hirschman’s functional knee phenotype classification [11]. These parameters were categorized into coronal (n = 9), sagittal (n = 7), and axial (n = 7) groups based on the imaging planes employed.

### 2.1. Coronal Parameters

**Figure 1:**
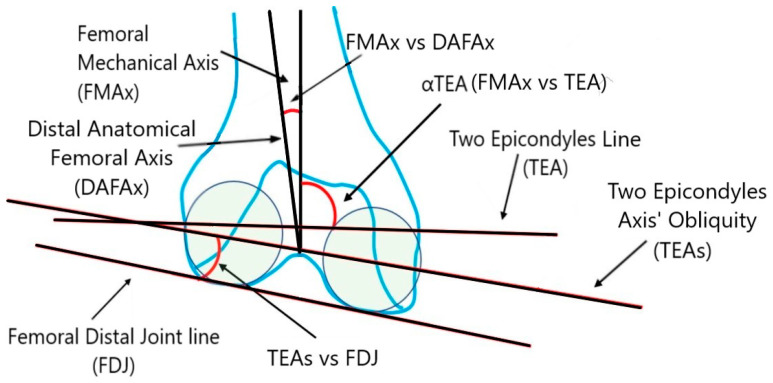
Graphical representation of coronal morphometric—provided as additional material.

**Figure 2:**
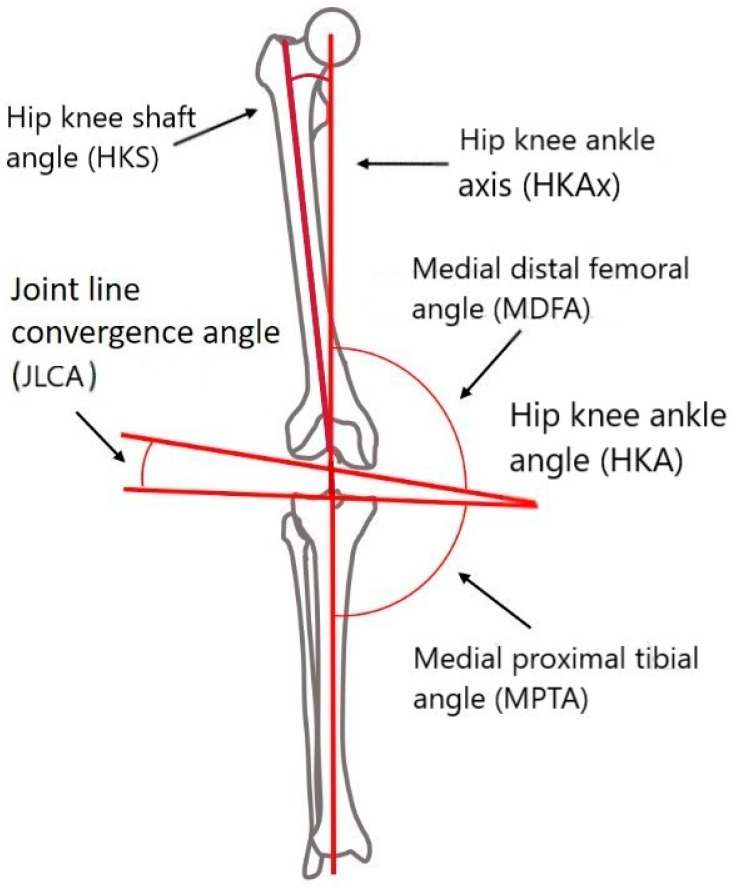
Graphical representation of additional coronal morphometric.

The parameters on the coronal plane encompass a variety of angles and axes, including the hip–knee–ankle angle (HKA), the medial proximal tibial angle (MPTA), the medial distal femoral angle (MDFA), the hip–knee–shaft angle (HKS), the angle between the femoral mechanical axis and the two epicondyles’ axis (α TEA), the joint line convergence angle (JLCA), the angle between the two epicondyles’ axis obliquity and the femoral distal joint line (TEAs vs. FDJ), the angle between the tibial mechanical axis and the proximal anatomical tibial axis (TMAx vs. PATAx), and the angle between the femoral mechanical axis and the distal anatomical femoral axis (FMAx vs. DAFAx) [Figure 1 and Figure 2]. The alignments of the femur and tibia’s joint lines were assessed concerning their mechanical axes. The MDFA and MPTA were determined as follows [Figure 2]: MDFA was gauged as the medial angle between the FMAx and a tangent to the distal femoral condyles. Meanwhile, MPTA was assessed as the medial angle between the TMAx and a tangent to the proximal tibial joint surface (tibial plateau). The choice to measure the femoral joint line orientation medially contrasts with Paley et al.’s suggestion of measuring the lateral angle (mechanical lateral distal femoral angle [mLDFA]). However, opting to measure both angles (DFA and PTA) medially presents greater coherence. A measurement surpassing 90° in MDFA or MPTA signifies a varus alignment of the femur or tibia, respectively.

### 2.2. Sagittal Parameters

**Figure 3:**
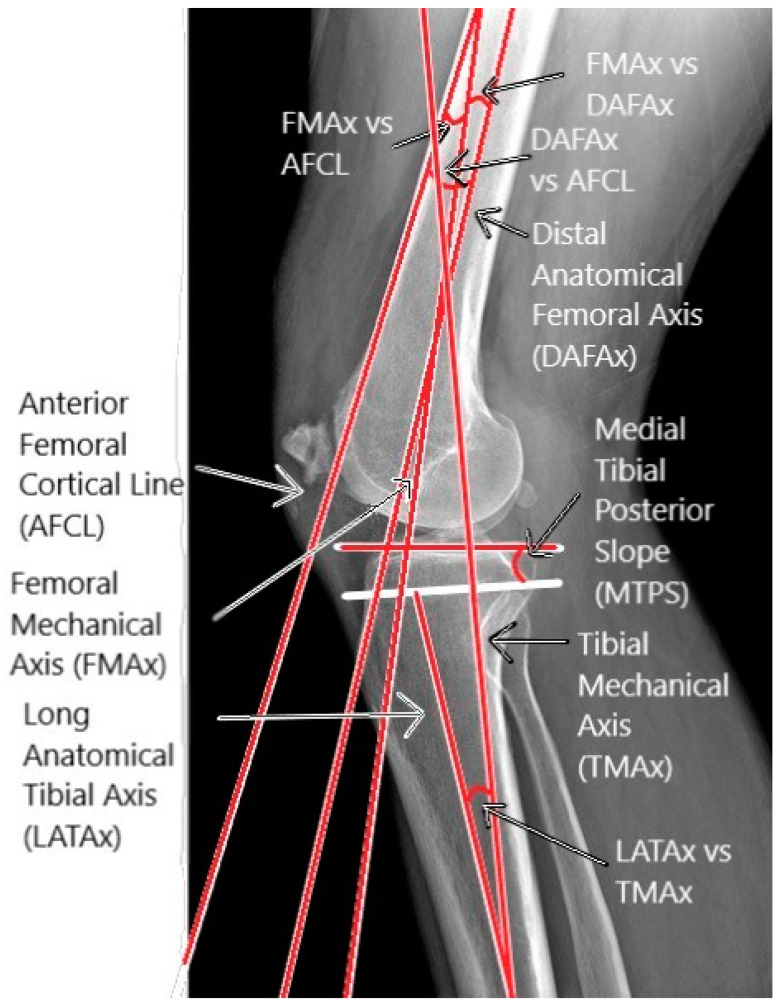
Graphical representations of sagittal morphometric.

The analyzed sagittal plane parameters are represented by the HKA angle in the sagittal plane (sagittal HKA), the angle between FMAx and the anterior femoral cortex line (FMAx vs. AFCL), the sagittal angle between FMAx and DAFAx (sFMAx vs. DAFAx), the angle between AFCL and DAFAx (AFCL vs. DAFAx), the medial tibial posterior slope (MTPS), the lateral tibial posterior slope (LTPS), and the angle between the long anatomical tibial axis and TMAx (LATAx vs. TMAx) [Figure 3].

### 2.3. Axial Parameters 

**Figure 4:**
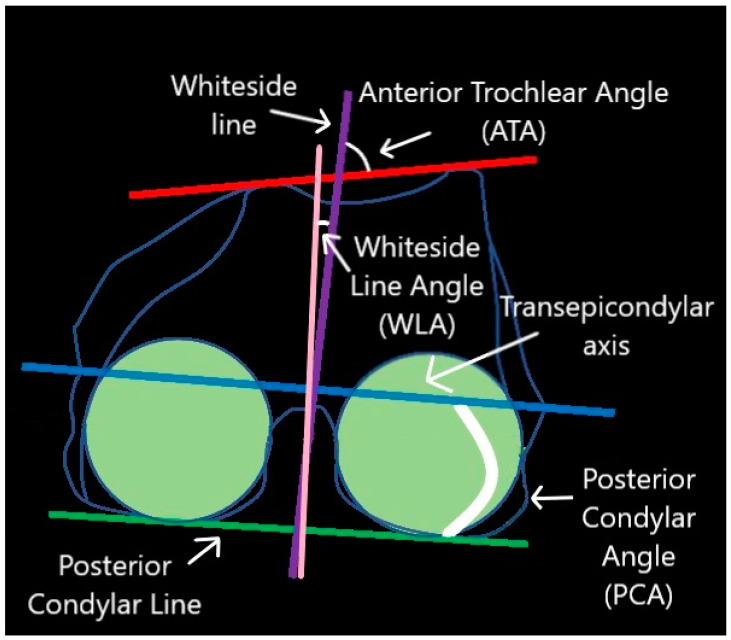
Graphical representation of axial morphometric parameters.

**Figure 5:**
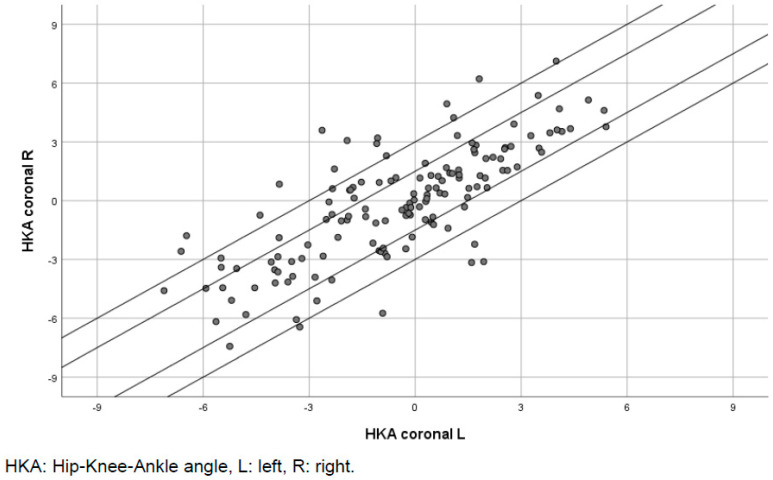
Left and right coronal knee phenotypes distribution with scatter plots and defined margins.

In the case of the axial plane, the following parameters were analyzed: the posterior femoral mechanical angle (FMA post), the posterior condylar angle (PCA), the anterior trochlear angle (ATA), the Whiteside line angle (WLA), femoral anteversion (AVF), external tibia torsion (ETT) and femoro-tibial rotation (F-T-Rot) [Figure 4 and Figure 5]. The FMA post has been analyzed with the knee in question bended. PCA has been calculated as the angle between the posterior cruciate ligament (PCL) and the two epicondyles’ axis (TEA). ATA represents the angle between the anterior trochlear line (ATL) and TEA. AVF has been attained through calculating the angle between PCL and the femoral neck shaft axis. Finally, the F-T-Rot has been noted as the angle between the TEAs and the medio-lateral axis (ML) of the proximal tibia in the axial plane. 

### 2.4. Statistical Analysis 

Pearson correlations were utilized to compare angles between the left and right knees, examine phenotype similarities, and assess standard deviation percentages. A power analysis for our sample size of N = 141 resulted in correlations of r = 0.24 with 80% power and r = 0.27 with 90% power. However, correlations below 0.5 were deemed irrelevant for this project. Subsequently, confidence intervals for Pearson correlations were tested to ensure the validity of our hypotheses (Table 1). The analyses were conducted using IBM SPSS 26.0 for Windows. Due to the uneven gender distribution (90 males, 51 females), separate analyses were conducted for each gender to account for potential differences in knee angles. Paired sample *t*-tests were employed to evaluate differences between the left and right legs, with effect sizes interpreted using Cohen’s d. According to Cohen’s classification, a d-value of 0.2 signifies a “small” effect size, 0.5 represents a “medium” effect size, and a value of ≥0.8 indicates a “large” effect size. 

## 3. Results

### 3.1. Coronal Parameters

- The highest correlated similarities (Pearson’s r = 0.76, 0.68, 0.76, 0.76, *p* < 0.001) between the left and right knees were seen in HKA (−0.48° ± 2.75° vs. −0.16° ± 2.89°), MDFA (93.15° ± 1.99° vs. 93.58° ± 1.95°), MPTA (87.32° ± 2.26° vs. 87.14° ± 2.46°) and HKS (4.94° ± 0.7° vs. 4.86° ± 0.66°). Conversely, notable systematic differences from left to right were observed in certain analyzed coronal parameters, reaching statistical significance (*p* < 0.05). The largest effect size was found between the left and right TMAx vs. PATAx (0.94° ± 1.43° [Cohen’s d = 0.66, t = 7.8, *p* < 0.001]). A detailed overview regarding the rest of the analyzed coronal parameters can be found in Table 2. A visualization of the left and right coronal knee phenotypes distribution with scatter plots and defined margins can be found in Figure 5.
- When discussing Hirschmann’s coronal knee phenotypes classification, in the case of HKA, 58% of patients (n = 82) displayed the same HKA phenotypes in both their knees. In the case of MDFA, the proportion of study subjects that displayed the same phenotype in both their knees is slightly higher, with 64% (n = 90). Additionally, 57% of the patients (n = 81) exhibited left and right knee MPTAs that belong in the same phenotype. Despite taking all these factors into account, only 26% of the patients (n = 37) exhibited identical Hirschmann’s coronal knee phenotypes in both knees. More than 2/3 of the patients (67% [n = 94]) have their knees in adjacent coronal knee phenotypes, when compared left to right (Table 3).

### 3.2. Sagittal Parameters 

The most significant similarity observed between the left and right knees was in the sagittal HKA (−10.04° ± 6.36° vs. −10.03° ± 6.53° [Pearson’s r = 0.92, *p* < 0.001]). Additionally, both MTPS and LTPS exhibited strongly correlated similarities between the knees (7.05° ± 3.27° vs. 7.24° ± 3.28° [Pearson’s r = 0.72, *p* < 0.001]; 6.89° ± 3.35° vs. 7.45° ± 3.47° [Pearson’s r = 0.64, *p* < 0.001]). Further details on the other analyzed sagittal parameters can be found in Table 2.

### 3.3. Axial Parameters

- The highest correlated similarities (Pearson’s r = 0.43, 0.44, 0.43, *p* < 0.001) between the left and right knees were seen in FMA post (91.76° ± 1.79° vs. 92.1° ± 1.64°), F-T-Rot (5.55° ± 4.57° vs. 5.54° ± 4.17°) and PCA (1.75° ± 1.81° vs. 2.06° ± 1.65°).
- Conversely, the statistical analysis revealed multiple noteworthy (*p* < 0.001) systematic differences between the left and right knees, specifically concerning WLA (2.78° ± 4° [Cohen’s d = 0.70, t = 8.26, *p* < 0.001]), ETT (−3.39° ± 7.01° [Cohen’s d = 0.48, t = −5.75, *p* < 0.001]) and ATA (−1.31° ± 3.1° [Cohen’s d = 0.42, t = −5.03, *p* < 0.001]). A detailed overview regarding the rest of the analyzed axial parameters can be found in Table 2.

## 4. Discussion

The pivotal findings of the study were as follows:

Firstly, although, 58% of patients (n = 82) presented identical coronal HKA phenotypes in both their legs, 64% (n = 90) showed the same MDFA phenotype and 57% (n = 81) showed the same MPTA phenotype, only 26% (n = 37) showed the same combined functional knee phenotype in both knees. However, 67% (n = 94) were in adjacent combined coronal functional phenotypes. Additionally, strongly correlated left-to-right symmetries have been identified in the coronal plane, in the form of HKA, HKS, MDFA and MPTA. Secondly, a very clear and strong symmetry was also found in the sagittal plane between left and right sagittal HKA. Additionally, several axial parameters displayed moderately correlated left-to-right similarities (i.e., FMA post, F-T-Rot, PCA and MPTS). Conversely, one of the most interesting findings is the presence of important systematic differences between several morphometric parameters; in particular, in the coronal and axial planes, between left and right TMAx vs. PATAx, WLA, ETT and ATA. These systematic differences have been shown to be as high as ~3° in the case of ETT and WLA. 

The findings indicate a reasonable left-to-right symmetry in all planes, which may play a role in the selection of bone cuts, soft tissue releases and the placement of implant components during TKA. However, given that deviations from the theoretical ideal alignment can lead to implant instability, high rates of revision and increased wear [14,16], the fact that the remaining analyzed parameters did not show strongly correlated left-to-right similarities, and that furthermore, several important systematic differences have been found, should also be taken into account. Therefore, the previously stated hypothesis has been partially proved. Although the existence of several similarities but the lack of a strict symmetry between knees has been predicted by the authors of this study, the presence of systematic differences has been unexpected. 

In the realm of knee morphology studies, various investigations have delved into the left–right symmetry of native knees, focusing on the coronal, axial, or sagittal planes [12,13,14,15]. However, this study marks a significant departure, standing as the first non-cadaveric analysis that meticulously scrutinized an extensive array of morphometric parameters (n = 23) across all three planes. This comprehensive scope allowed for a more nuanced understanding of knee alignment and morphology, offering insights into the intricacies of left–right symmetry within each plane. When exploring left–right symmetry in the axial plane, our findings align with those of Eckhoff et al., corroborating the existence of correlated similarities between the left and right knees [13]. This validation lends weight to the notion of consistent bilateral characteristics in axial knee parameters, emphasizing the stability and reproducibility of these observations. In the sagittal plane, our study’s identification of correlated similarities between left and right LTPS and MTPS findings echoes the work of Jacquet et al., reinforcing the notion of dependable bilateral patterns within sagittal knee parameters [14]. It also underscores the reliability of these observations across varied study settings, fortifying, up to a certain point, the concept of bilateral symmetry in sagittal knee morphometry. Regarding the coronal plane, our results pertaining to HKA, MPTA, and MDFA find resonance with the prevailing literature [13,14,15]. Notably, Beckers et al. conducted a study comparing coronal HKA, the tibial mechanical angle (TMA), the femoral mechanical angle (FMA), and functional knee phenotypes between the left and right knees using long-leg standing X-rays. Their findings echoed our observations to an extent, indicating different percentages of paired knees exhibiting similar functional knee phenotypes [12]. This might be due to the different data gathering modality. In the aforementioned study, long-leg radiographs instead of SPECT/CT were used. 

However, despite the wealth of research on knee symmetry in individual planes, no previous study has thoroughly explored systematic differences between left and right morphometric parameters across the coronal, sagittal, and axial alignment of native knees. Our comprehensive evaluation and identification of systematic differences in various morphometric parameters not only enriches the understanding of bilateral knee symmetry but also highlights the need for a more nuanced approach to interpreting knee morphometry. This nuanced approach could potentially inform better clinical decision-making, especially in procedures such as total knee arthroplasty (TKA) or osteotomies, where precise alignment plays a pivotal role in patient outcomes.

There are a number of limitations to this study. Firstly, a possible selection bias needs to be acknowledged. In addition, the relative low number of enrolled patients could have significantly influenced the results. A small sample size increases the risk of Type II errors, where true effects are not detected due to insufficient statistical power. All the study participants were Europeans. Therefore, the present findings might not completely apply to the populations of other continents [28,29]. Although the knees were evaluated in three planes, only for the coronal one could a validated classification be used, making the assessment of knee alignment in the sagittal and axial planes less standardized, potentially affecting the comprehensiveness of the findings. Additional phenotypes for the sagittal and axial planes are necessary for a more inclusive classification of these alignments. Additionally, this study has been based entirely on native knees. Consequently, the clinical importance (in the case of OA knees) is somewhat limited. However, it has been suggested before that the morphology of OA knees may bear a strong connection to the morphological characteristics of native, healthy knees [30]. Moreover, it has also been determined that certain features of the knee joint, such as the shape of the tibial plateau and femoral condyles, can be used to predict the development and progression of OA [30]. While this area of research is still evolving, these models have the potential to improve our understanding of OA. Furthermore, artificial intelligence-driven predictive models also may play a role in improving the early detection and diagnosis of OA, as well as inform personalized treatment strategies for patients with OA [31].

## 5. Conclusions

Only 26% of native knees exhibit an identical coronal phenotype in their contralateral knee, whereas 67% have the adjacent coronal phenotype. Strongly correlated resemblances were established across various left and right knee morphometric parameters in the coronal, sagittal, and axial planes. These findings could enhance decisions in procedures like total knee arthroplasties or osteotomies, where alignment is key to outcomes, and reveal a potential for future artificial intelligence-driven models to improve our understanding and improve personalized treatment strategies for knee osteoarthritis. 

## Figures and Tables

**Table 1:** Intervals of confidence of Pearson r for N = 141.

R	0.20	0.30	0.40	0.50	0.60	0.70	0.80	0.90
CI low	0.04	0.14	0.25	0.36	0.48	0.60	0.73	0.86
CI high	0.35	0.44	0.53	0.61	0.69	0.78	0.85	0.93

CI: Confidence interval.

**Table 2:** Similarities and systematic differences between left to right knee morphometric parameters.

Variable	LEFT Mean +/− SD	RIGHT Mean +/− SD	Pearson r	*p*	Mean Diff. +/− SD	t	Cohen’s d	*p*
HKA coronal	−0.48 +/− 2.75	−0.16 +/− 2.89	0.76	0.000	−0.32 +/− 1.94	−1.98	0.17	0.049
MDFA	93.15 +/− 1.99	93.58 +/− 1.95	0.68	0.000	−0.43 +/− 1.59	−3.22	0.27	0.002
MPTA	87.32 +/− 2.26	87.14 +/− 2.46	0.76	0.000	0.19 +/− 1.66	1.33	0.11	n.s.
FMA post	91.76 +/− 1.79	92.1 +/− 1.64	0.43	0.000	−0.34 +/− 1.84	−2.18	0.18	0.031
JLCA	−0.95 +/− 1.4	−0.92 +/− 1.33	0.55	0.000	−0.02 +/− 1.29	−0.22	0.02	n.s.
HKA sagittal	−10.04 +/− 6.36	−10.03 +/− 6.53	0.92	0.000	−0.02 +/− 2.64	−0.07	0.01	n.s.
HKS	4.94 +/− 0.7	4.86 +/− 0.66	0.76	0.000	0.08 +/− 0.47	2.14	0.18	0.034
TEAs vs. FMAx	7.55 +/− 2.23	7.8 +/− 2.15	0.74	0.000	−0.25 +/− 1.58	−1.89	0.16	n.s.
TEA vs. FDJ	1.37 +/− 1.12	1.39 +/− 1.29	0.29	0.001	−0.02 +/− 1.44	−0.17	0.01	n.s.
AVF	17.65 +/− 8.54	16.28 +/− 8.48	0.64	0.000	1.37 +/− 7.2	2.27	0.19	0.025
ETT	28.92 +/− 8.84	32.32 +/− 8.65	0.68	0.000	−3.39 +/− 7.01	−5.75	0.48	0.000
F−T−Rot	5.55 +/− 4.57	5.54 +/− 4.17	0.44	0.000	0.01 +/− 4.63	0.03	0.00	n.s.
FMAx vs. AFCL	−2 +/− 8.51	−0.32 +/− 3.12	0.28	0.001	−1.68 +/− 8.2	−2.44	0.21	0.016
FMAx vs. DAFAx (coronal)	3.45 +/− 2.58	3.92 +/− 2.07	0.63	0.000	−0.47 +/− 2.06	−2.70	0.23	0.008
AFCL vs. DAFAx	5.45 +/− 8.76	4.24 +/− 3.2	0.32	0.000	1.22 +/− 8.33	1.73	0.15	n.s.
FMAx vs. DAFAx (sagittal)	4.29 +/− 1.47	4.73 +/− 1.57	0.59	0.000	−0.44 +/− 1.38	−3.78	0.32	0.000
MTPS	82.95 +/− 3.27	82.76 +/− 3.28	0.72	0.000	0.19 +/− 2.44	0.94	0.08	n.s.
LTPS	83.11 +/− 3.35	82.55 +/− 3.47	0.64	0.000	0.56 +/− 2.89	2.32	0.20	0.022
LATAx vs. TMAx	2.25 +/− 0.73	2 +/− 0.69	0.61	0.000	0.24 +/− 0.63	4.63	0.39	0.000
TMAx vs. PATAx	0.17 +/− 1.7	−0.77 +/− 1.9	0.69	0.000	0.94 +/− 1.43	7.80	0.66	0.000
PCA	1.75 +/− 1.81	2.06 +/− 1.65	0.43	0.000	−0.31 +/− 1.85	−2.00	0.17	0.047
ATA	5.31 +/− 3.3	6.63 +/− 3.58	0.60	0.000	−1.31 +/− 3.1	−5.03	0.42	0.000
WLA	3.6 +/− 3.58	0.81 +/− 4.23	0.49	0.000	2.78 +/− 4	8.26	0.70	0.000

SD: Standard Deviation; n.s: not significant; Diff: Difference; HKA coronal: Hip-Knee-Ankle coronal angle; MDFA: Medial Distal Femoral Angle; MPTA: Medial Proximal Tibial Angle; FMA post: Femoral Mechanical Angle posterior; JLCA: Joint Line Convergence Angle; HKA sagittal: Hip-Knee-Ankle sagittal angle; HKS: Hip-Knee-Shaft angle; TEAs vs. FMAx: Angle between the two epicondyles and the Femoral Mechanical Axis; TEA vs. FDJ: Angle between the two epicondyles’ line obliquity and the Femoral Distal Joint line obliquity in the coronal plane; AVF: Femoral Anteversion; ETT: External Tibia Torsion; F-T-Rot: Femorotibial torsion; FMAx vs. AFCL: Angle between Femoral Mechanical Axis and Anterior Femoral Cortex Line; FMAx vs. DAFAx (coronal): Coronal angle between Femoral Mechanical Axis and Distal Anatomical Femoral Axis; AFCL vs. DAFAx: Angle between Anterior Femoral Cortex Line and Distal Anatomical Femoral Axis; FMAx vs. DAFAx (sagittal): Sagittal angle between Femoral Mechanical Axis and Distal Anatomical Femoral Axis; MTPS: Medial Tibia Posterior Slope; LTPS: Lateral Tibia Posterior Slope; LATAx vs. TMAx: Angle between Long Anatomical Tibia Axis and Tibial Mechanical Axis; TMAx vs. PATAx: Angle between Tibial Mechanical Axis and Proximal Anatomical Tibia Axis; PCA: Posterior Condylar Angle; ATA: Anterior Trochlear Angle; WLA: Whiteside Line Angle.

**Table 3:** Comparison of coronal phenotypes.

Phenotype	HKA		MPTA		MDFA		Coronal Phenotype	
	N	%	N	%	N	%	N	%
Same	82	58.2	90	63.8	81	57.4	37	26.2
Adjacent	51	36.2	50	35.5	58	41.1	94	66.7
Greater difference	8	5.7	1	0.7	2	1.4	10	7.1

HKA: hip–knee–angle; MDFA: medial distal femoral mechanical angle; MPTA: medial proximal tibial angle.

## Data Availability

All data supporting the reported results can be found in the Kantonnspital Baselland’s registry and can be accessed upon personal inquiry to the corresponding author.
